# Current Surgical Options for Moyamoya Disease

**DOI:** 10.7759/cureus.11332

**Published:** 2020-11-04

**Authors:** Julie Mayeku, Miguel A Lopez-Gonzalez

**Affiliations:** 1 Neurosurgery, Loma Linda University Medical Center, Loma Linda, USA

**Keywords:** moyamoya, ischemic stroke, hemorrhagic stroke, cerebral bypass, sta-mca bypass, revascularization

## Abstract

Moyamoya disease (MMD) is a cerebrovascular disease of unknown etiology characterized by stenotic and occlusive arterial changes of the anterior circulation, with subsequent proliferative development of arterial collateralization. In spite of there being limited understanding of the clear etiology of MMD, surgical revascularization for MMD is considered the standard treatment to prevent further stroke. While the use of surgical revascularization to prevent future hemorrhagic stroke in MMD is still controversial, it is considered effective in the case of ischemic stroke. This article presents a review of the current surgical management of MMD based on an analysis of the most recent data from peer-reviewed articles and opinion based on personal experience with surgical revascularization in the treatment of MMD.

## Introduction and background

Moyamoya disease (MMD) is a chronic cerebrovascular disorder of unknown etiology characterized by stenotic and occlusive arterial changes in particular of the anterior cerebral circulation. These chronic ischemic changes lead to the proliferation of the collateral arterial network, which is evident with the classic “puff of smoke” appearance on cerebral angiograms. While the clear etiology and pathogenesis of MMD is not completely understood, recent studies show that the incidence and prevalence of MMD have increased [1-4], although part of this can be related to clinicians being more aware of the disease and with more neuroimaging options to make a diagnosis. The bimodal age distribution usually occurs with one peak in the first decade of life and a second one in the fourth decade, and clinical manifestations can be with either hemorrhagic or ischemic stroke [1-2]. Besides the focal ischemic symptoms, other nonfocal symptoms to consider are headaches, vertigo, dizziness, syncope, seizures, and cognitive decline. Children seem to present more often with ischemic symptoms. Arias et al. report that approximately 79% of the children with MMD present with ischemic stroke symptoms [1]. MMD is a progressive disease with an annual stroke rate of 13.3%, leading to recurrent strokes in patients [5-6], although some patients show stability in their symptoms.

There are currently no stroke preventive measures in MMD or proven medical therapy [1,6]. The main objectives of surgical revascularization in ischemic MMD involve augmenting the cerebral blood flow, controlling symptoms, and preventing future ischemic strokes [1,4,6-7] while in hemorrhagic MMD disease, the hypothesis of bypass benefit is decreasing the long-term hemodynamic stress in collateral vessels, thus decreasing the recurrence of hemorrhages [8].

Augmenting the intracranial blood flow improves the resting cerebral blood flow and vascular reserve capability. In effect, the blood supply is restored and cerebrovascular hemodynamics stabilized. This leads to the regression of the fragile moyamoya vessels, thus preventing bleeding. With successful revascularization, additional stroke prevention and improvement in neurological and cognitive outcomes are expected. Revascularization can be achieved surgically - by direct revascularization, indirect revascularization, or a combination of both. There is currently no consensus on the ideal type of revascularization [5]; nonetheless, the advantages and disadvantages of individual techniques will be further discussed in this article.

## Review

Indications for surgical revascularization procedures

For the past few decades, several studies have established the use of revascularization surgery for symptomatic MMD as the standard treatment for preventing further strokes [1-2]. The main purpose of surgical revascularization is to decrease recurrent ischemic or hemorrhagic strokes as well as to improve the neurological functions of patients. In particular, the effectiveness of revascularization surgery as a treatment of ischemic MMD has been well-established by various studies. In patients with ischemic MMD, hemodynamic disturbances, rather than thromboembolic events, are considered to be the principal cause of the symptoms. Surgical procedures are, therefore, used to augment cerebral blood flow (CBF) in a distal vascular territory to the stenotic and occlusive areas, thus decreasing the risk of further ischemic episodes.

The use of surgical revascularization for hemorrhagic MMD is nowadays more accepted to prevent rebleeding in hemorrhagic MMD. We should point out that a few studies have suggested that the use of surgical revascularization procedures in adult hemorrhagic MMD could be beneficial. For instance, the Japan Adult Moyamoya (JAM) trial showed that the direct bypass technique could prevent rebleeding in adult hemorrhagic MMD [8], and subsequent analysis found that posterior hemorrhages had a higher risk of rebleeding than anterior vascular territories and achieve greater benefit with surgery [9]. While these findings support the use of bypass surgery to treat patients with hemorrhagic disease, there is currently no known treatment that is capable of reversing the disease process [1,3,8,10-11].

Commonly accepted indications for surgical revascularization include the following: clinical symptoms for either ischemic or hemorrhagic strokes with decreased cerebral blood flow, vascular response, and perfusion reserve [1,3,12]. There are three different revascularization methods used in the surgical management of MMD, namely, direct revascularization, indirect revascularization, and combined revascularization. There is generally no agreement on the best type of revascularization surgery, nonetheless, this review focuses on investigating and analyzing the effectiveness of the different revascularization strategies for the management of MMD [1].

Types of surgical revascularization

Direct Revascularization

Direct revascularization is a well-established technique with excellent reported clinical outcomes. The first successful direct revascularization using the superficial temporal artery to middle cerebral artery (STA-MCA) bypass for MMD was performed by Yasargil in 1967. To date, it remains one of the most precise microsurgical procedures ever performed. Of course, given the current advancement in technology, there have been various modifications to the original procedure [7].

**Figure 1:**
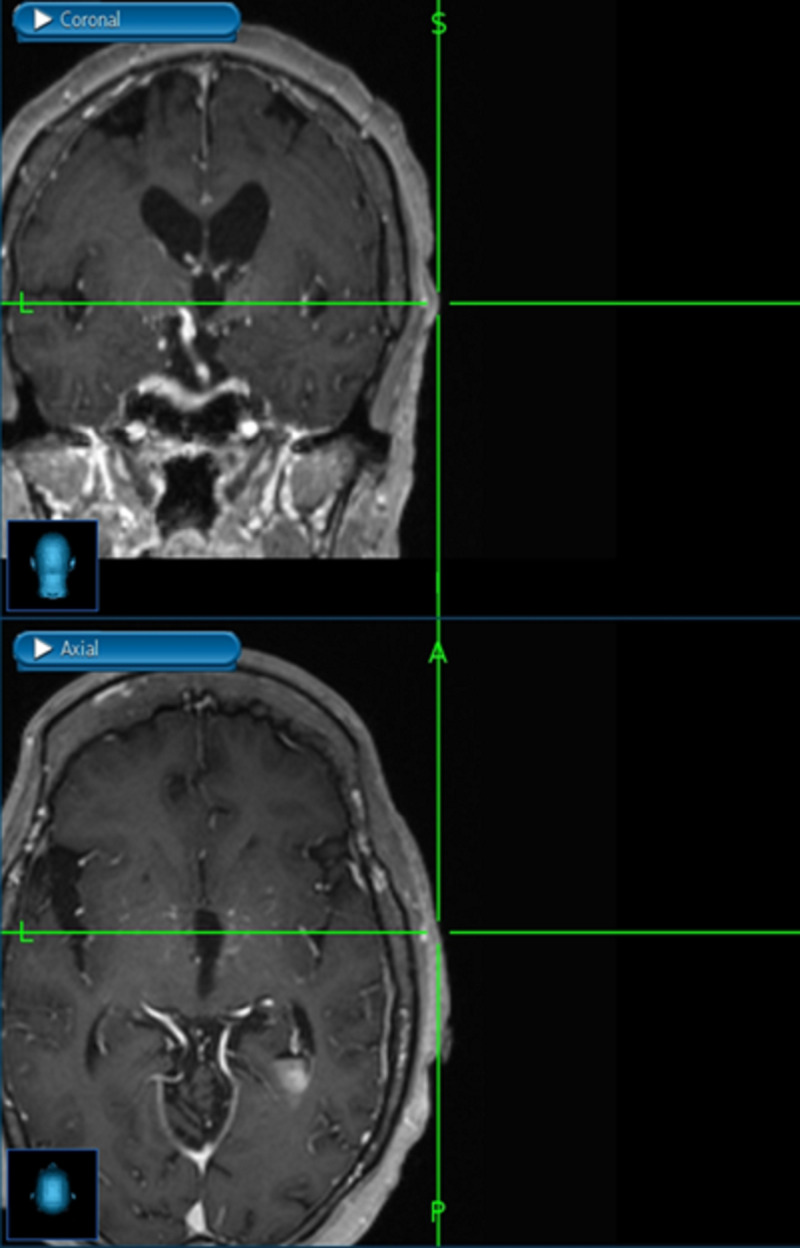
Stereotactic navigation to target craniotomy around the Sylvian fissure

**Figure 2:**
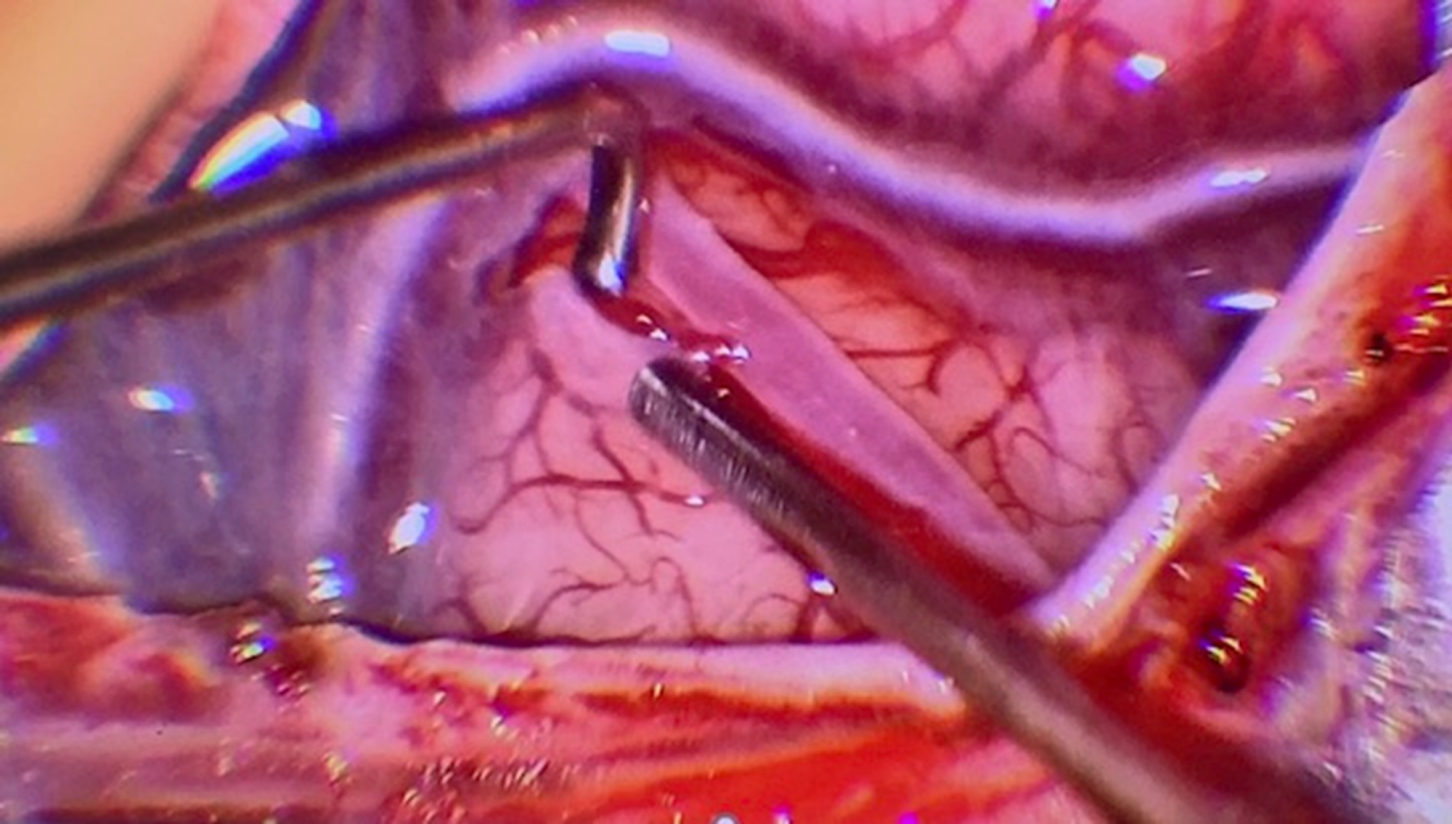
Dural opening with the craniotomy centered around the Sylvian fissure, exposing the M4 branch of the middle cerebral artery

**Figure 3:**
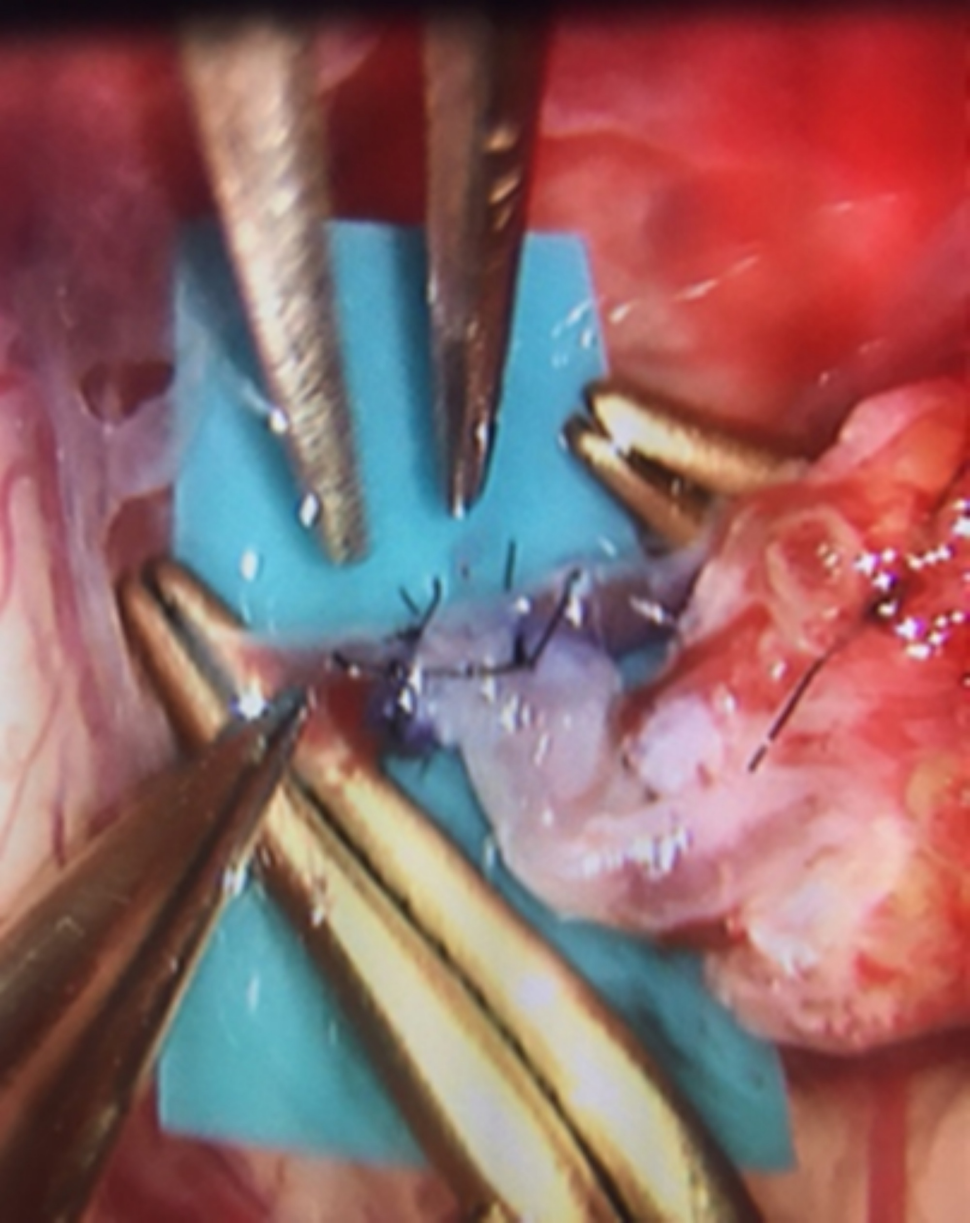
Intraoperative view of direct STA-MCA revascularization STA-MCA: superficial temporal artery to middle cerebral artery

In the direct revascularization technique, the STA is mainly used as the donor vessel to be anastomosed to a distal branch of the MCA. The STA has the frontal and parietal branch, where either one or both can be used as donor vessels and can be traced by surface Doppler and neuronavigation. It is important to mention that the ideal angiographic diameter should be equal or larger than 1 mm, although not to be discouraged with smaller sizes since, not infrequently, a smaller angiographic STA is larger on surgical dissection and measurement and carries adequate flow for anastomosis. The dissection of the STA should be gentle to avoid vasospasm or injury, leaving a small cuff of soft tissue around the vessel, and the cut flow measured (usually 30-60cc/min). The distal end of the STA is dissected clean of galea tissue, irrigated with heparinized saline after the proximal STA clipping, and prepared for anastomosis. Another warning, when possible, it is important to preserve one of the STA branches in order to decrease the chance of scalp necrosis. Additionally, combined bypass requiring a larger incision than direct bypass is associated with skin healing issues (15.2% vs 3%) while the type of incision is also relevant since lineal incision was found to have less risk of healing complications (1.6%) than curved (3.8%) or Y-type of incisions (17.1%) [13]. Besides the common STA-MCA bypass, it is less common to perform anastomosis to the anterior cerebral artery (ACA) or posterior cerebral artery (PCA) territories. In the rare case of requiring ACA territory revascularization, a large STA segment (at least 10 cm) should be harvested with targeted craniotomy to the ACA territory. In the event of performing both MCA and ACA territory revascularization, both STA branches need to be harvested and two separate craniotomies need to be planned; ideally, one targeted around the Sylvian fissure and a second one toward the ACA territory, passing the longer STA donor vessel underneath the bone bridge between both craniotomies to perform the anastomosis near the midline. It is important to mention that anytime both STA branches are harvested, the potential risk for scalp healing issues can be higher. However, the occipital artery (OA) can also be used to revascularize either the MCA or PCA territory. It must be noted that posterior quadrant direct revascularization is more challenging, in particular, for smaller recipient artery sizes, as well as more difficult to dissect in case of the use of the occipital artery [3,11-12]. The average recipient vessel diameter is 1 mm, and it can be performed even in 0.8 or 0.9 mm vessels. When the craniotomy is performed and dura exposed, it is important to preserve middle meningeal branches, and open the dura around those, since its collaterals not infrequently have distal cortical anastomosis, which can be evaluated on preoperative conventional angiogram. On direct revascularization, it is our preference to perform stereotactic craniotomy targeted at the level of the Sylvian fissure (Figure 1), where adequate recipient cortical vessels can be found (Figure 2), and with the objective to perform small access and minimize morbidity without compromising the exposure (Figure 3).

If the cortical branches of the MCA are not large enough, then a Sylvian fissure dissection can be used to reach an adequate recipient vessel. During the dissection of the cortical recipient vessel, our preference is to avoid any coagulation of small branches while placing the color background for the anastomosis in order to decrease the chances for ischemic complications. The anastomosis is performed with interrupted 10-0 monofilament sutures with a taper point needle. After the removal of temporary clips, the flow should be confirmed with micro-Doppler and indocyanine green intravenous injection. Any problems to confirm adequate patency should be fixed immediately, revising the donor graft, or including the anastomosis site. Few interrupted sutures are easily removed, if needed, to revise the anastomosis site, which can be more challenging while using a running suture.

**Figure 4:**
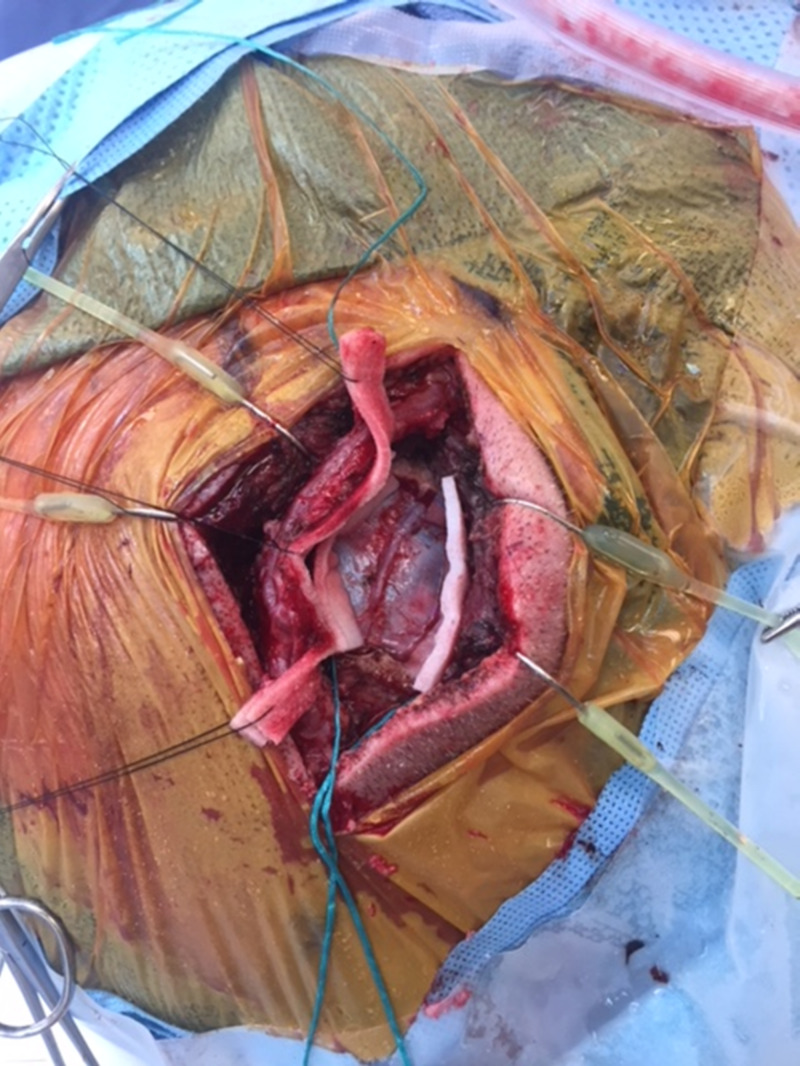
Intraoperative minimal invasive incision and craniotomy

**Figure 5:**
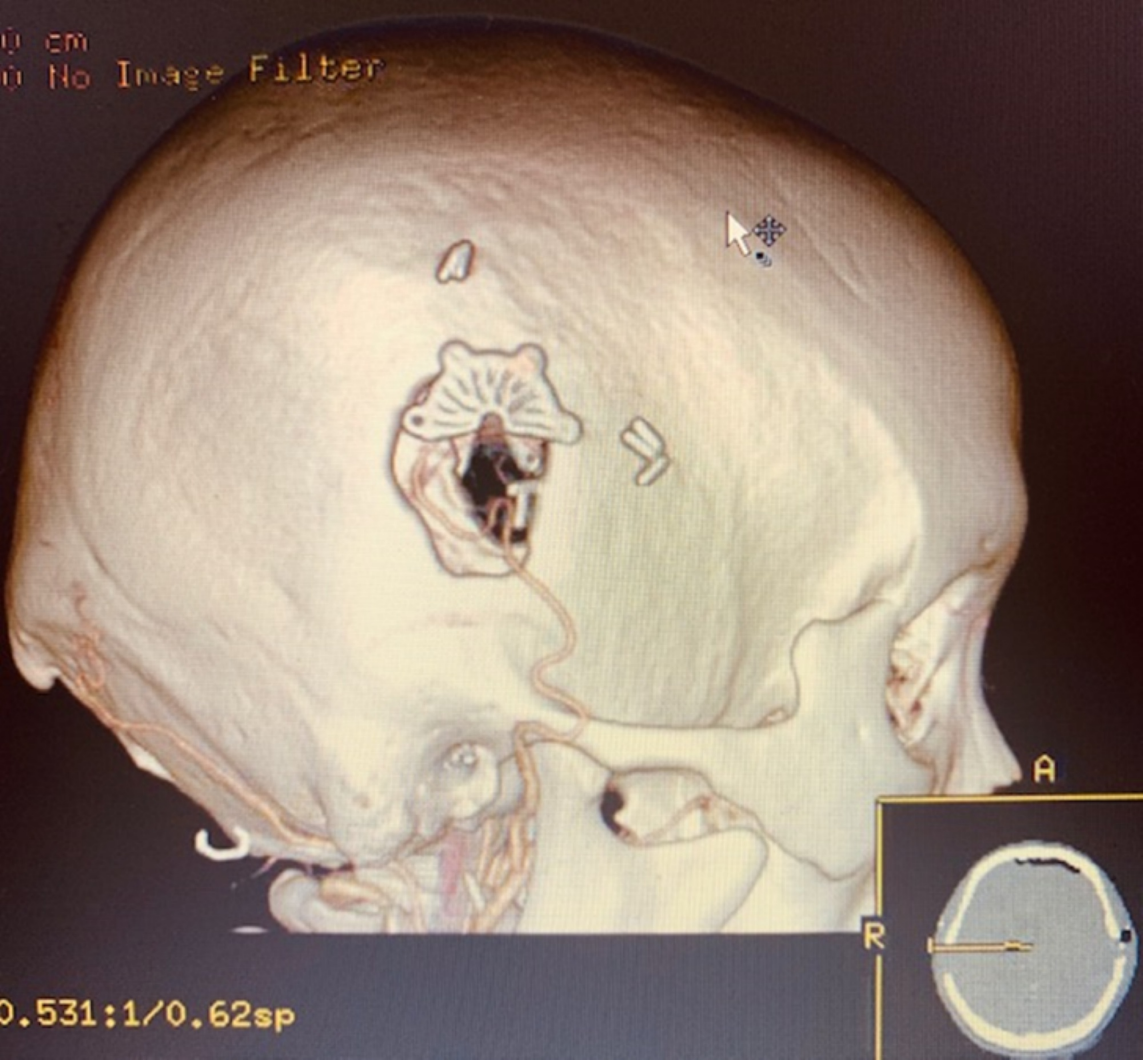
Immediate postoperative CT angiogram confirming direct bypass anastomosis through a minimally invasive approach

A major advantage of direct revascularization is that it demonstrates its immediate effects by improving the cerebral perfusion in the surgical area postoperatively, which can also be performed through a minimally invasive approach (Figures 4-5)

The downside of direct revascularization in pediatric patients, in particular, was small or fragile vessels precluding adequate anastomosis in the late Suzuki stage [3,6].

Perioperatively, aspirin is used before and after surgery, and is important to avoid hypotension, hyperventilation, or the use of mannitol. Electrophysiology monitoring is recommended during surgery, and graft patency is evaluated with CTA in the immediate postoperative period. On subsequent follow-up, CTA and MRI perfusion is recommended yearly.

Currently, direct revascularization leads the other techniques as the most well-established revascularization surgery with immediate improvement in cerebral hemodynamics outcomes in patients. It also reports more patients with improved clinical symptoms and decreased future stroke events compared with conservative management with an odds ratio of 0.301 (P<0.001) based on meta-analysis only since no randomized clinical trial exists for ischemic MMD [5]. In a recent randomized control trial, a reduced risk of hemorrhagic stroke was reported as an additional benefit in MMD patients who underwent direct bypass as compared to patients who had conservative therapy for MMD management [2].

Direct procedures have the following disadvantage: the technique is inherently more challenging, requiring greater technical expertise with extensive micro-anastomosis training and experience. As mentioned earlier, it also requires the availability of suitable donor or recipient vessels, especially in children. Overall, the morbidity rate has been reported at 3.5% while mortality is at 0.7% [13]. Direct re-vascularization involves slightly higher early postoperative risks. For instance, Steinberg et al. have reported a 5% to 15% risk of transient neurological deficits postoperatively, even without negative diffusion-weighted imaging (DWI) changes on MR imaging. This can possibly be attributed to acute changes in blood flow. A direct bypass can also lead to radiological hyperperfusion (incidence between 18% and 50%) that can progress to hemorrhage complications in approximately 1.7% [3,13]. Other less common risks but not negligible are bypass occlusion (less than 2%), which can be multifactorial and surgical technique-dependent, scalp-healing issues (1.7% to 5.1%) [14], seizures, and subdural hematoma, for which patients should be well informed [13].

Indirect revascularization

Indirect revascularization techniques rely on the ingrowth of new blood vessels by bringing additional blood flow through vascularized tissue and promoting the development of new vessels. Unlike direct bypass, indirect bypass does not use vessel anastomosis. There are different technique options for indirect revascularization, including, but not limited to, encephaloduroarteriosynangiosis (EDAS), encephalomyosynangiosis, multiple burr holes, encephalo-duro-myo-arterio-pericranial synangiosis, and omentum transplantation. Which of all these options is superior to the others is not yet known [1,3,6]. Overall, it depends on institutional preference and experience while it is important to consider a large craniotomy size for a wider surface area to promote collateral network formation, specifically for encephaloduroarteriosynangiosis and encephalo-duro-myo-arterio-pericranial synangiosis. 

Indirect revascularization is ideal for patients lacking an adequate size of the recipient vessels to perform the direct anastomosis such as pediatric patients or in rare adult cases of advanced-stage MMD. These techniques are evidently easier than direct bypass procedures, with a shorter intraoperative time and hence reduced intraoperative and/or postoperative complications. Additionally, due to the low flow rate of indirect revascularization procedures, hyperperfusion syndrome is rarely experienced postoperatively.

The small size of cortical branches of the distal PCA limits the use of direct bypass in the temporal posterior-parietal-occipital areas, making indirect bypass a good alternative for revascularization for strokes in the PCA territories. Additionally, the occipital artery can also be used in indirect bypass procedures similar to EDAS [6]. Indirect revascularization has notably been very effective in children compared to adults. The higher success rate of indirect revascularization in children may be attributed to higher cerebrovascular plasticity at younger ages [3,6-7]. In this context, Deng et al. report 40%-50% of failed neovascularization in adult patients [3].

A major disadvantage of the indirect bypass as compared to direct revascularization is that cerebral neovascularization may take months to develop. Collateral development following indirect bypass is estimated to take three to four months. Immediate revascularization should, therefore, not be expected in patients undergoing indirect revascularization alone. Indirect bypass procedures are also less predictable, with reported incomplete collateralization in some cases leading to inconsistencies in revascularization outcomes [3-4,7].

Combined revascularization

Combined revascularization refers to the use of both the direct and indirect bypass techniques concurrently for the management of MMD. Combined bypass aims at obtaining an immediate improvement of cerebrovascular hemodynamics as a result of direct bypass, along with additional benefits from the diffuse neovascularization expected to develop over time from the indirect bypass. Consequently, indirect bypass aids in further improving revascularization results as well as adding a back-up strategy if the direct anastomosis is insufficient or fails. While this line of thought has generally been entertained by clinicians, leading to increased use of combined bypass in the management of MMD, a quantitative assessment of the results show unpredictable outcomes across individual patients [15]. While several studies report the advantages of using direct bypass over indirect bypass in managing patients with MMD, it is unclear whether the use of direct bypass alone has any clear advantage in clinical outcomes over the use of combined bypass in the management of MMD, despite the former’s angiographic superiority [3,11,14]. According to Cho et al. [2], combined bypass should be performed in adult MMD patients symptomatic from MCA and ACA regions instead of complex direct bypass surgery. Additionally, the benefit of a combined bypass in the pediatric population has also been found, although the subject of superiority of direct versus indirect versus combined bypass is still a matter of ongoing controversy [16]. A certain disadvantage of the combined revascularization method is the relatively larger area required to perform the dissection and surgical exposure.

It is relevant to acknowledge that a small percentage of patients with persistent ischemic symptoms post-revascularization might require additional revascularization due to inadequate arterial collateralization [9]. If new infarcts are present on follow-up neuroimaging studies, or the presence of persistent poor cerebrovascular reserve, repeat revascularization can be considered. According to Steinberg et al., the majority of repeat revascularizations for the prior direct bypass group were done to augment blood supply in a different vascular territory. Over half of these were achieved using an additional direct bypass technique. In their study, Steinberg et al. concluded that repeat revascularization can be safely performed and can effectively prevent future ischemic events [4].

## Conclusions

In conclusion, while it is clear that open surgical revascularization for MMD is superior as compared to medical management or observation, the ideal revascularization option remains controversial. Each one of the techniques carries inherent advantages and disadvantages. Direct or combined revascularization in symptomatic adults seems to be favorable, preventing stroke, while indirect revascularization appears more effective in pediatric patients. Ideally, more prospective data would be helpful to determine the best surgical option in patients with MMD. Until then, adequate patient selection, surgeons’ experience and skills, as well as intra and postoperative care, play a major role in the success of the procedure, reflected in favorable patient outcomes.
